# Elapid Snake Venom Analyses Show the Specificity of the Peptide Composition at the Level of Genera *Naja* and* Notechis*

**DOI:** 10.3390/toxins6030850

**Published:** 2014-02-28

**Authors:** Aisha Munawar, Maria Trusch, Dessislava Georgieva, Diana Hildebrand, Marcel Kwiatkowski, Henning Behnken, Sönke Harder, Raghuvir Arni, Patrick Spencer, Hartmut Schlüter, Christian Betzel

**Affiliations:** 1 Laboratory of Structural Biology of Infection and Inflammation, Institute of Biochemistry and Molecular Biology, University of Hamburg, c/o DESY, Notkestreet 85, Building 22a, Hamburg 22603, Germany; E-Mails: aishamunawar_2001@yahoo.com (A.M.); octopus_dofleini@yahoo.com (D.G.); 2 Department of Chemistry, University of Engineering & Technology, G.T. Road, Lahore 54890, Pakistan; 3 Institute of Organic Chemistry, University of Hamburg, Martin-Luther-King-Platz 6, Hamburg 20146, Germany; E-Mails: trusch@chemie.uni-hamburg.de (M.T.); behnken@chemie.uni-hamburg.de (H.B.); 4 Institute of Clinical Chemistry, University Medical Centre Hamburg-Eppendorf (UKE), Martinistraße 52, Hamburg 20246, Germany; E-Mails: d.hildebrand@uke.de (D.H.); m.kwiatkowski@uke.de (M.K); soeharder@gmx.de (S.H.); hschluet@uke.de (H.S.); 5 Department of Physics, IBILCE/UNESP, Rua Cristóvão Colombo 2265, São José do Rio Preto CEP 15054-000, SP Brazil; E-Mail: arni@sjrp.unesp.br; 6 Centro de Biotecnologia, Instituto de Pesquisas Energéticas e Nucleares, Avenue Lineu Prestes 2242, São Paulo 05508-000, Brazil; E-Mail: pspencer@IPEN.BR

**Keywords:** *Naja mossambica mossambica*, *Notechis scutatus* from Kangaroo Island, pharmacologically active peptides, snake venom, cytotoxin, neurotoxin, natriuretic peptides, Kunitz-type inhibitor, bradykinin-potentiating peptides

## Abstract

Elapid snake venom is a highly valuable, but till now mainly unexplored, source of pharmacologically important peptides. We analyzed the peptide fractions with molecular masses up to 10 kDa of two elapid snake venoms—that of the African cobra, *N. m. mossambica* (genus *Naja*), and the Peninsula tiger snake, *N. scutatus*, from Kangaroo Island (genus *Notechis*). A combination of chromatographic methods was used to isolate the peptides, which were characterized by combining complimentary mass spectrometric techniques. Comparative analysis of the peptide compositions of two venoms showed specificity at the genus level. Three-finger (3-F) cytotoxins, bradykinin-potentiating peptides (BPPs) and a bradykinin inhibitor were isolated from the *Naja* venom. 3-F neurotoxins, Kunitz/basic pancreatic trypsin inhibitor (BPTI)-type inhibitors and a natriuretic peptide were identified in the *N.* venom. The inhibiting activity of the peptides was confirmed *in vitro* with a selected array of proteases. Cytotoxin 1 (P01467) from the *Naja* venom might be involved in the disturbance of cellular processes by inhibiting the cell 20S-proteasome. A high degree of similarity between BPPs from elapid and viperid snake venoms was observed, suggesting that these molecules play a key role in snake venoms and also indicating that these peptides were recruited into the snake venom prior to the evolutionary divergence of the snakes.

## 1. Introduction

Elapid snake venoms contain a large number of pharmacologically active peptides, influencing important physiological functions, like blood coagulation and the cardiovascular and nervous systems [1]. Venom peptides are a rich and potent source of the prototypes of novel drugs. Their high target specificity, structural stability, relative ease of chemical synthesis and the possibility to improve the drug efficacy by chemical modifications are very suitable for pharmaceutical application and for the design of novel medicines. In this connection, the partnership of the Australian biopharmaceutical company, QRxPharma Ltd (North Sydney, Australia), and its subsidiary, Venomics Pty Ltd (VPL) (North Sydney, Australia), with the University of Queensland for the development of novel drug prototypes from elapid snake venom is a remarkable example of collaboration between science and the pharmaceutical industry. As a result, three novel compounds acting on the hemostatic system are in preclinical development [2,3]. There exists a number of other examples of the successful application of the knowledge about venom peptide structure and function for pharmaceutical purposes. Probably the most impressive example is the development of the well-known anti-hypertensive drug, Captopril^® ^ [4] and other derivatives [5]—angiotensin-converting enzyme (ACE) inhibitors designed on the basis of the *B. jararaca* venom peptide structure. The necessity of new generation medicines and the application of the venom peptide structure for drug design are discussed in a review published recently [6]. Venom peptides are a novel alternative to a number of contemporary existing drugs. For this reason, further studies on the snake venom peptidome are of pharmaceutical and clinical significance. At present, there is a demand to develop a new generation of anti-hypertensive drugs without or with lesser side effects. This can be achieved by selective blocking of one of the two domains of ACE [7]. Snake venom peptide structures can serve as models for respective drug design investigations. The crystal structure of BPPb (snake venom bradykinin-potentiating peptide, a selective inhibitor of the C-domain of ACE) in complex with the C-domain of human ACE illustrates that the inhibitor binds at the active site in a Zn-independent manner, revealing new modes of active site interactions compared to the so far described ACE-inhibitor complex structures [8].

This study describes a comparative venom peptide analysis of two elapid snakes: *Naja mossambica mossambica* (representative of the genus, *Naja*) and *N.* *scutatus* from Kangaroo Island (genus *Notechis*). Both snakes are widely recognized species of the genera, *Naja* and *Notechis*. *Naja* species belong to the most widespread group of snakes known as cobras, found in Africa and Asia. *Notechis* (tiger snakes) is a large group of snakes distributed in Australia. They are among the most venomous in the world [9]. 

## 2. Results

### 2.1. Purification and Identification of Peptides from N. m. mossambica Venom

**Figure 1:**
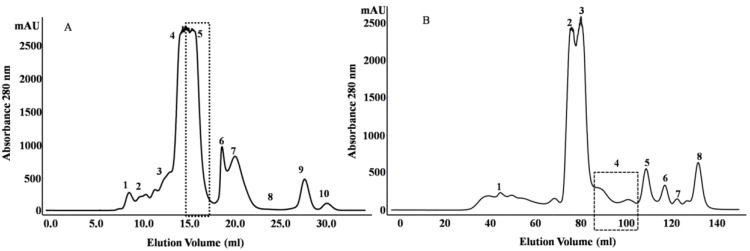
(**A**) Size-exclusion chromatography of *N. m. mossambica* venom on a Superdex C-75 10/300 column at pH 5.0; (**B**) size-exclusion chromatography of the *N. scutatus* from Kangaroo Island venom on a Superdex G-75 16/60 column at pH 5.0.

**Figure 2:**
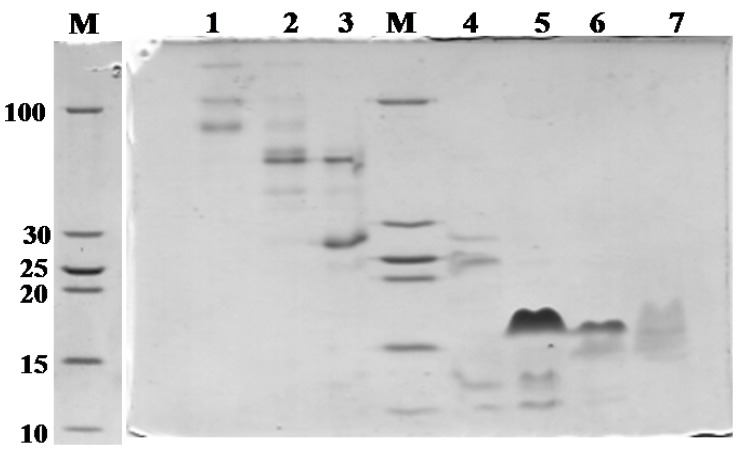
SDS-PAGE (Sodium dodecyl sulfate-polyacrylamide gel electrophoresis) of the fractions, 1–7, from the size exclusion chromatography of *N. m. mossambica* venom.

**Figure 3:**
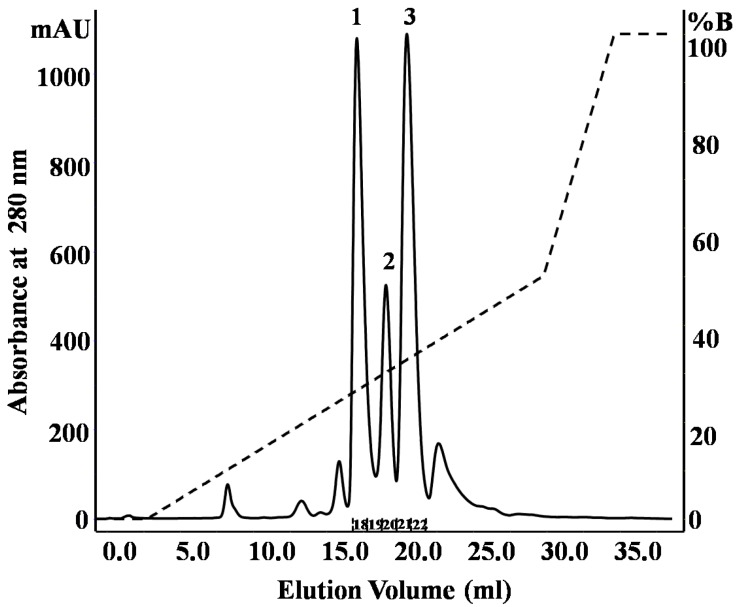
Further purification by fast protein liquid chromatography (FPLC) of Peak 5 (Figure 1A) with a Resource-S column (1 mL) at pH 5.5.

Figure 1A shows the fractionation of the *N. m. mossambica* venom. SDS-PAGE (Sodium dodecyl sulfate-polyacrylamide gel electrophoresis) demonstrated the presence of peptides below 10 kDa in Peaks 4–10 (Figure 2). Peak 5 showed inhibitory activity towards subtilisin (StmPr1), chymotrypsin and trypsin. The fractions marked as 8 and Peak 10 showed inhibitory activity towards ACE. Peak 5 was further purified with a Resource S column at pH 5.5 by liquid chromatography (Figure 3), and three main peaks were observed. Fraction 18 showed inhibitory activity towards subtilisin, chymotrypsin and trypsin. Matrix-assisted laser desorption/ionization time of flight mass spectrometry (MALDI-TOF-MS) and electrospray ionization time of flight mass spectrometry (ESI-TOF-MS) showed the presence of a 6819.28 Da peptide in the first peak. The MALDI-TOF mass spectrometric analysis showed the presence of peptides with molecular masses of 6726 Da and 6837 Da in Peaks 2 and 3, respectively.

Purification of the peptides from Peak 5 (Figure 1A) is described in detail to illustrate the approach used for the purification of peptides from *N. m. mossambica* venom. The same procedure was adopted to isolate and characterize the peptides listed in Table 1. A peptide with a molecular mass of 6895.4 Da was identified in Peak 6 and peptides with masses of 872.5–1276.6 Da were identified in Peaks 8 and 10. 

**Table 1:** Pharmacologically active peptides isolated from the *Naja mossambica mossambica* venom. Abbreviations used: ACE, angiotensin-converting enzyme; BPP, bradykinin-potentiating peptide; Z, pyroglutamyl residue; X, isoleucine/leucine; SEC, size-exclusion chromatography. The molecular masses of the peptides were determined by matrix-assisted laser desorption/ionization time of flight mass spectrometry (MALDI-TOF-MS) or electrospray ionization time of flight mass spectrometry (ESI-TOF-MS). The larger peptides were digested by trypsin and the resulting peptides analyzed by LC/ESI-ion trap MS. The identified tryptic peptides are shown in bold. Small peptides were directly analyzed by MALDI-TOF/TOF MS.

Fraction number (SEC)	Observed m/z	Score/sequence coverage	Sequence	Inhibitory activity	Homology with peptide from	Peptide family
5	975.1823 (M + H)^7+^	274/70%	LKCNQLIPPFWKTCPKGKNLCYKMTMRAAPMVPVKRGCIDVCPKSSLLIKYMCCNTNKCN	Bacterial subtilisin (StmPr1), chymotrypsin and 20S proteasome	P01467: *Naja mossambica*	Snake venom three-finger toxin (CTX M1)
5, 6	6896.40 (M + H)^+^	71/48%	LKCNRLIPPFWKTCPEGKNLCYKMTMRLAPKVPVKRGCIDVCPKSSLLIKYMCCNTNKCN		P01470: *Naja mossambica*	Snake venom three-finger toxin (CTX M3)
5	6727.402 (M + H)^+^	119/93%	LKCNKLIPIAYKTCPEGKNLCYKMMLASKKMVPVKRGCINVCPKNSALVKYVCCSTDRCN		P01452: *Naja mossambica*	Snake venom three-finger toxin (CTX M4)
8	873.4511 (M + H)^+^	32	ZQKFSPR		P85314: *Bothrops moojeni*	Zinc metalloproteinase
8	901.4614 (M + H)^+^	30	ZQRFSPR		Q072L5: *Bothrops asper*	Zinc metalloproteinase/disintegrin
8	779.3983 (M + H)^+^	22	ZXWPRP	ACE	P0C7R6: *Agkistrodon piscivorus piscivorus*	BPP
10	1214.6581 (M + H)^+^	51	ZXWPRPQXPP	ACE	P0C7S6: *Crotalus atrox*	BPP
10	1276.6380 (M + H)^+^	45	ZQWPPGHHXPP	ACE	P0C7J9: *Crotalus adamanteus*	BPP
10	1063.5611 (M + H)^+^	20	TPPAGPDVGPR		Q27J49: *Lachesis muta muta*	Bradykinin inhibitor peptide
10	1230.6743 (M + H)^+^	18	QXWPRPQXPP	ACE	P0C7S6: *Crotalus atrox*	BPP
10	1277.6380 (M + H)^+^	26	ZEWPPGHHXPP	ACE	Q27J49: *Lachesis muta muta*	BPP

**Figure 4:**
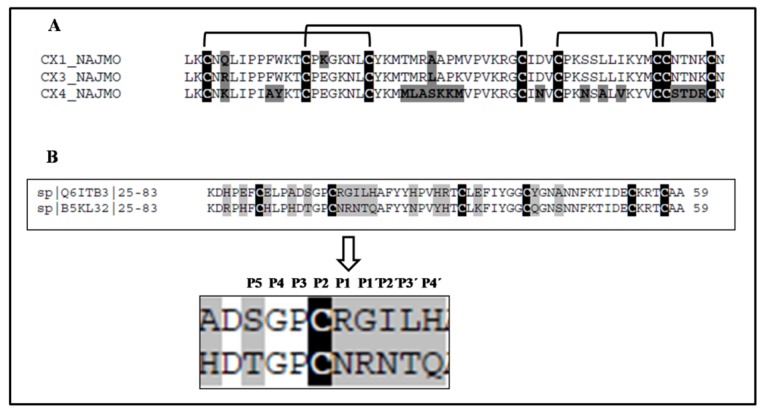
(**A**) Sequence alignment of cytotoxin 1 (P01467), cytotoxin 3 (P01470) and cytotoxin 4 (P01452) isolated from *N. m. mossambica* venom. The cysteine residues are shaded black, and the disulfide bonds are indicated. The variable amino acid residues are shaded grey; (**B**) the sequence alignment of the Kunitz inhibitors isolated from the *N. scutatus* (Kangaroo Island) venom shows a variation in the reactive site residues. The same peptides, named protease inhibitor tigerin 1 (Q61TB3) and tigerin 3 (B5KL32), were found by transcriptome analysis in the *Notechis scutatus scutatus* venom gland [10].

Three peptide cytotoxins (cardiotoxins), cytotoxin 1 (P01467, CTX M1), cytotoxin 3 (P01470, CTX M3) and cytotoxin 4 (P01452, CTX M4) were identified in the *N. m. mossambica* venom (Table 1). The cytotoxins were identified by analysis of tryptic digests using LC/ESI ion trap MS and subsequent database search. Fraction 18 of Peak 1 (Figure 3) contained cytotoxin 1 (P01467), Peak 2, cytotoxin 4, and Peak 3 was a mixture of cytotoxins 1 and 3. Cytotoxin 3 (P01470) was also identified in Peak 6 (Figure 1A). The identified fragments matching the toxin sequences are shown in bold in Table 1. Figure 4A shows a sequence alignment of the three cytotoxins. The different amino acid residues are shaded grey, and the cysteine residues are highlighted in black. Cytotoxins 1 and 3 show 93% sequence identity with each other. The three cytotoxins have different amino acids at positions 5 and 28. Lys 16 of cytotoxin 1 (P01467) is substituted by glutamic acid in the other two toxins. 

**Figure 5:**
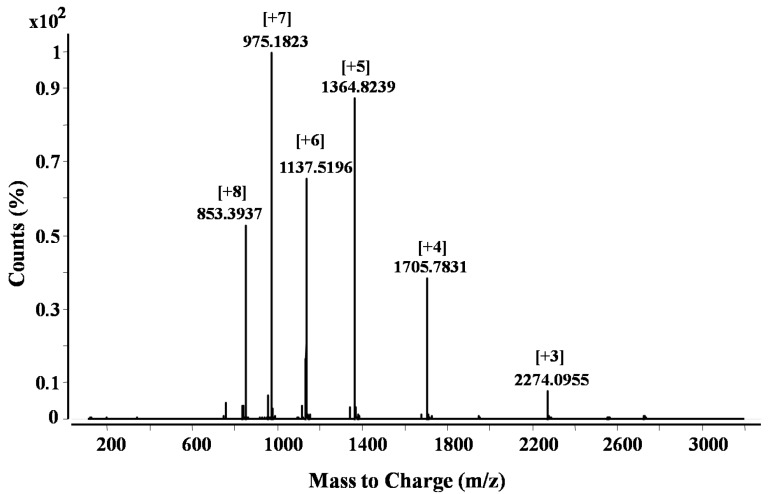
ESI-TOF-MS spectrum of cytotoxin-1, P01467 (isolated from *N. m. mossambica* venom) showing multiply charged ions.

CTX M4 shows 68% sequence identity in comparison to the other two cytotoxins. The differences in amino acids are in the three loop regions. CTX M1 and CTX M3 have proline at position 30, and therefore, they belong to the P-type cytotoxins, while CTX M4 has SER 28 and can be classified as an S-type cytotoxin [11]. The differences in amino acid residues suggest the functional diversity of these polypeptides. Fraction 18 of Peak 1 (Figure 3) containing CTX M1 inhibited subtilisin (StmPr1) and chymotrypsin, while CTX M3 did not inhibit any of the tested enzymes. CTX M1 was also found to inhibit the chymotryptic-like activity of the 20S proteasome strongly. From the ESI-TOF-MS spectrum (Figure 5), the molecular weight of cytotoxin 1 (P01467) in fraction 18 (Figure 3) was calculated to be 6819.3 Da. 

**Figure 6:**
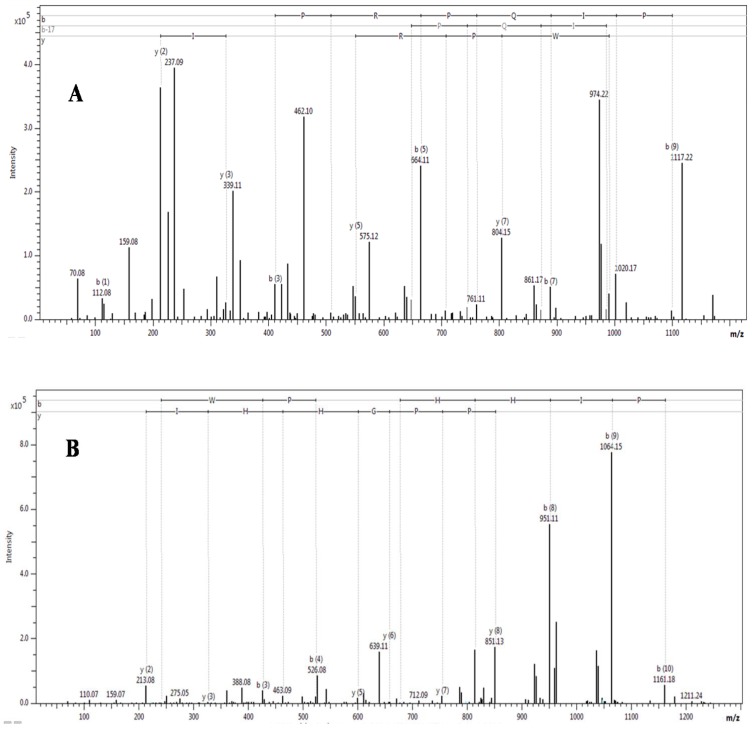
MALDI TOF/TOF MS of the representative peptides isolated from *N. m. mossambica*. (**A**) At m/z 1214.6581 (M + H)^+^; (**B**) at m/z 1276.6380 (M + H)^+^.

Eight single charged ions of m/z 873–1277 (M + H)^+^ were also identified in *Naja m. mossambica* venom (Table 1). The sequences of all peptides were determined by MALDI-TOF/TOF mass spectrometry. The MALDI-TOF/TOF of representative peptides with m/z 1214.6581 (M + H)^+^ and m/z 1276.6380 (M + H)^+^ is shown in Figure 6. The sequence annotation pictures of the MALDI-TOF/TOF spectra were prepared with the Bruker software ProteinScape, version 3.0. Five of these peptides belong to the BPP family. One bradykinin inhibitor peptide, previously isolated from *Lachesis muta muta* and *Agkistrodon bilineatus* venoms, was also identified. The other two peptides are small fragments of a snake venom metalloproteinase. The peptide, ZXWPRPQXPP, is a modified form of QXWPRPQXPP, which was isolated from the *Crotalus atrox* venom*,* with the N-terminal Q modified to pyroglutamate. ZEWPPGHHXPP and ZQWPPGHHXPP are homologous peptides with a single amino acid substitution. ZXWPRP is another form of ZXWPRPQXPP lacking the C-terminal QXPP.

### 2.2. Purification and Identification of Peptides from N. scutatus (Kangaroo Island) Venom

**Figure 7:**
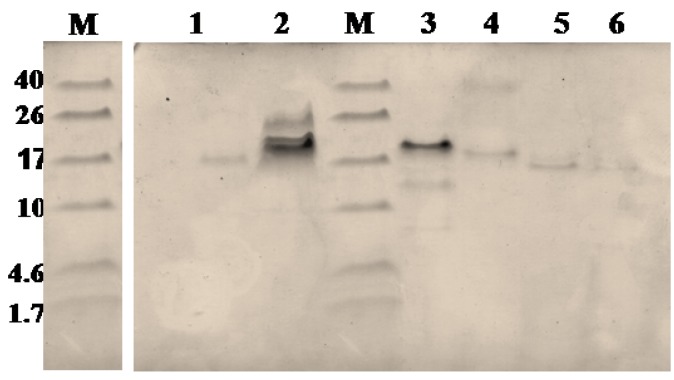
SDS-PAGE of fractions 1–6 from the size exclusion chromatographic separation (Figure 1B) of the crude venom of *N. scutatus* (Kangaroo Island).

**Figure 8:**
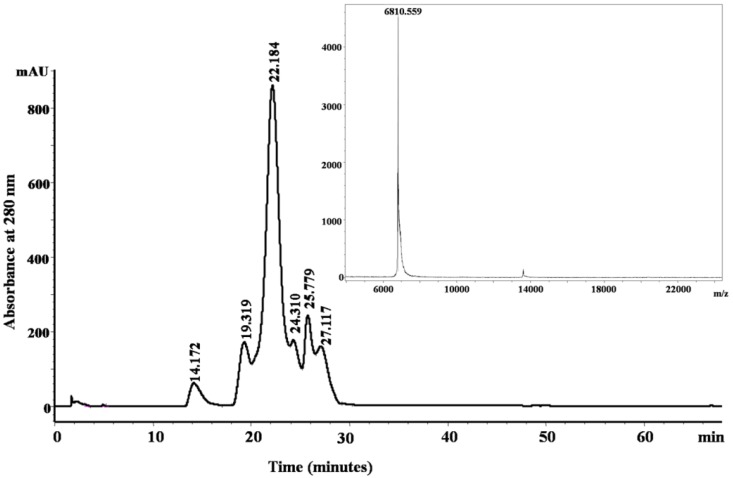
Purification of Peak 4 (Figure 1B) with a SOURCE™ 5RPC column by HPLC. Inset shows MALDI-TOF-MS of the peptide (tigerin-3, B5KL32) eluting at 19.319 min.

Figure 1B shows the fractionation of the* N. scutatus* venom. The peaks were analyzed by SDS-PAGE (Figure 7) and the fractions containing peptides with molecular masses <10 kDa (Peaks 4–8) were collected for further analysis. Inhibitory activities towards trypsin, plasma kallikrein, plasmin and angiotensin-converting enzyme were found in Peak 4. These fractions were pooled and the peptides separated by reverse phase chromatography (Figure 8). Samples were subjected to MALDI-TOF-MS and tryptic digest analysis. The inset of Figure 8 shows the MALDI-TOF-MS of fraction eluting at 19.3 min. The sequences and observed masses of the peptides are summarized in Table 2. The peptides identified by tryptic digestion are shown in bold. The data allowed the classification of the peptides from both snakes into protein/peptide families using three indexes: molecular masses, enzyme inhibitory activity and amino acid sequences. Two short neurotoxins with molecular masses of 6647.9 Da and 6687.7 Da (Table 2) were purified by reverse phase chromatography. These neurotoxins have been reported previously at a transcript level in the venom of other Australian elapid snakes: *Notechis scutatus scutatus* and *Austrelaps superbus*, respectively [12]. They are 60-amino acid polypeptides with high homology. Two Kunitz/basic pancreatic trypsin inhibitor (BPTI) peptides (Table 2) were also identified. They are 59-amino acid polypeptides and have been reported before at the transcript level as tigerin 1 (Q61TB3) and tigerin 3 (B5KL32) in the venom gland of *Notechis scutatus scutatus* [10]. We found that tigerin 1 (Q61TB3) shows inhibitory activity towards trypsin and kallikrein and also weakly inhibits plasmin, while tigerin 3 (B5KL32) inhibits only trypsin. Both Kunitz-type peptides did not inhibit either factor Xa or thrombin. Sequence analysis showed that the residues at the reactive site are different in the two toxins (Figure 4B). A natriuretic peptide, NP, with a molecular mass of 3347.7 Da, having inhibitory activity towards ACE, was also identified. Low molecular weight peptides, with m/z in the range 600–1600 (M + H)^+^, were separated by reverse phase chromatography. Fractions containing low molecular weight peptides with m/z in the range 600–1600 (M + H)^+^ strongly inhibited ACE. The molecular mass range suggests that they are probably novel BPPs. The MALDI-TOF/TOF MS analysis and subsequent database search by the Inhouse Mascot server did not retrieve any sequences. The ESI-QTOF-MS analysis of these peptide fractions showed the presence of glycopeptides. Signals were observed for the following glycan moieties: hexose, N-acetylhexosamine and N-acetylneuraminic acid. However, it was not possible to determine the peptide sequence of the glycopeptides, because the collision-induced dissociation technique fragments the carbohydrate moiety. The limited amount of material unfortunately did not allow for performing different mass spectrometric experiments, such as electron transfer dissociation, which would, in principle, provide the sequence information of the peptide, by fragmentation of the amino acids, and preserve the glycosylation on the peptide backbone [13]. Only one communication on the isolation of snake venom glycopeptides has been published so far: that on the venom composition of *Dendroaspis angusticeps* [14]. The isolated glycopeptides have a high proportion of proline residues with molecular masses ranging from 1.3 kDa to 2.4 kDa [14]. 

**Table 2:** Pharmacologically active peptides isolated from *N. scutatus* venom. Abbreviations used: ACE, angiotensin-converting enzyme; NP, natriuretic peptide; X, Leucine/isoleucine. All peptides were purified from Peak 4 of Figure 1B. The molecular masses of the peptides were determined by MALDI-TOF-MS. Tryptic peptides of larger peptides were analyzed by LC/ESI-ion trap MS. The peptides identified by tryptic digestion are shown in bold. Fractions containing glycopeptides were analyzed by ESI-Q-TOF MS.

Observed m/z (M + H)^+^	Score/sequence coverage	Sequence determined	Inhibitory activity	Homology with peptide from	Peptide family
6647.921^*^	185/44%	MTCCNQQSSQPKTTTTCAESSCYKKTWRDHRGTITERGCGCPNVKPGVQINCCKTDECNN		A8HDK0: *Notechis scutatus scutatus*	Snake venom three-finger toxin
6687.729	121/27%	MTCCNQQSSQPKTTTTCAESSCYKKTWRDHRGTIIERGCGCPNVKPGIQLVCCETNECNN		A8S6A4: *Austrelaps superbus*	Snake venom three-finger toxin
6810.559	1569/65%	KDRPHFCHLPHDTGPCNRNTQAFYYNPVYHTCLKFIYGGCQGNSNNFKTIDECKRTCAA	Trypsin	B5KL32: *Notechis scutatus scutatus*	Kunitz/BPTI
6694.233	231/45%	KDHPEFCELPADSGPCRGILHAFYYHPVHRTCLEFIYGGCYGNANNFKTIDECKRTCAA	Trypsin, plasma kallikrein, plasmin	Q6ITB3: *Notechis scutatus scutatus*	Kunitz/BPTI
3347.688	54/30%	SGSEVAKIGDGCFGLPLDRIGSASGMGCRSVPKP	ACE	Q3SAE8: *Notechis scutatus scutatus*	NP
1154.556		QNPPKPXSGES		Q3SAX8: *Oxyuranus scutellatus scutellatus*	NP
764.201 (M + H)^2+^					Glycopeptide
681.251					Glycopeptide

Notes:^ *^ The mass measured by us by MALDI mass spectrometry is 6647.921 (M + H)^+^, whereas the calculated one is 6639.4 Da. Clearly, there is a discrepancy between the calculated and the measured mass. The identity of A8HDK0 was obtained by analyzing tryptic peptides with LC-MS followed by a Mascot search. Since the Mascot score for the identification of A8HDK0 was reasonable, it can be assumed that this protein was present in the corresponding fraction. Several reasons are possible, also in combination, which may explain the discrepancy of the measured and calculated mass: 1. We obtained a sequence coverage of 44%. Thus, we do not have any information about the rest of the protein. The missing peptides may include posttranslational modifications, which also might explain why we missed these peptides in the search result of MASCOT. 2. An exchange of amino acids cannot be excluded. 3. Mistakes in the protein database can also not be excluded. The same explanation can be taken into account for other mass differences.

## 3. Discussion

Elapid species of the superfamily Colubroidea [15] are among the most toxic snakes of the world ([2,9] and the references therein). However, their venoms are poorly known from a toxicological and drug discovery point of view [2]. The analyses of the *N. m. mossambica* and *N. scutatus* venom peptide compositions revealed specificity at the genus level. The *Naja m. mossambica* venom peptidome contains cytotoxic-type three-finger toxins, bradykinin-potentiating peptides and a bradykinin inhibitor, but lacks Kunitz/BPTI inhibitors. At the same time, the peptide fraction analysis of *N. scutatus* venom revealed the presence of Kunitz/BPTI inhibitors, neurotoxic-type three-finger toxins and natriuretic peptides. Neurotoxins have been isolated from *N.* *m. mossambica*venom [16]. We did not find representatives of this group of toxins in the venom of the snake mentioned above. It is known that the venom composition depends on the diet of the snake, the geographical region, ontogenetic variations,* etc.* ([17] and the references therein). Furthermore, very low quantities of the respective toxins in the venom can prevent their identification. α-neurotoxins make up only 0.4%–12.6% of the venom proteins of the *Naja* species [18].

The* Naja* species possesses cardiotoxins, 60–62 residue basic peptides, pharmacologically distinct from the so-called “short neurotoxins” [19,20]. Cytotoxins predominate in *Naja* venom toxins and constitute 72.8% of the *Naja nigricollis* total venom proteins [18]. The three-finger toxins found in elapid venoms constitute a superfamily of non-enzymatic polypeptides, consisting of 60–74 amino acids [1,21]. Three-finger cardiotoxins have been reported exclusively in cobra venom. These toxins form ion pores in the lipid membranes [22]. The structure of CTXs was investigated by crystallographic [19,23] and NMR [24] methods. The cytotoxin 1 (P01467) that we have isolated from *Naja. m. mossambica* venom (Table 1) shows inhibitory activity towards the 20S proteasome, a 700 kDa multi-catalytic complex constituting the proteolytic core of the 26S proteasome complex. The complex possesses three active sites: chymotrypsin-like, trypsin-like and peptidyl-glutamyl peptide hydrolyzing (PGPH)-like ([25] and the references therein). Proteasomes are present in the cell nucleus and cytoplasm, and their main function is to maintain the concentration of specific proteins and to degrade damaged, oxidized or misfolded proteins [26]. The cells use ubiquitin-proteasome systems to maintain cellular homeostasis [27]. It can be supposed that after envenomation, the *Naja m. mossambica* venom cytotoxin 1 (P01467) might be involved in the disturbance of cellular processes by inhibiting a vital catalytic machinery of the cell, the 20S proteasome. Inhibition of this complex could be one of the mechanisms involved in cytotoxin-mediated cell death. Cytotoxin 1 (P01467) can serve as a promising candidate for cancer therapy, as the proteasome is an important target for the treatment of cancer [28,29]. Already, CTX III isolated from *Naja naja atra* venom was reported to have anticancer activity against human breast cancer cells [30].

The *N. scutatus* peptidome contains three-finger α-neurotoxins (Table 2). Neurotoxins are responsible for neurological effects as a result of envenomation by Australian snakes [12]. They inhibit nicotinic acetylcholine receptors (nAChRs) [12,17]. 

The snakes use venom hypotensive peptides to weaken their prey. Bradykinin-potentiating peptides are natural inhibitors of angiotensin converting enzymes, which play a key role in blood pressure regulation [5]. The pharmaceutical industry developed a large number of antihypertensive drugs, like captopril and other derivatives, designed using the BPP structure [7]. However the synthetic ACE inhibitors, used at present, cause serious side effects, which could be ascribed to their inability to discriminate between the C- and N- domains of the enzyme. C-domain-specific inhibitors could have the same effect as the currently available drugs, but with low or no side effects, due to decreased bradykinin and substance P levels [5]. The structure of novel BPPs, like that identified in *N m. mossambica* venom (Table 1), might help to design more specific C-domain ACE inhibitors, since this region of the enzyme is the major site of angiotensin-1 processing. It was demonstrated by Fry and Wüster [31] that snake venom toxins evolved from the recruitment of body proteins into the venom. Comparison of BPPs from elapid (this work) and viperid [32] snake venoms showed a high similarity, suggesting the recruitment of these peptides into the snake venom before the split of the two families. A possible reason for this is the important role of BPPs in prey immobilization. Hypotension is usually associated with poisoning upon snake envenomation [33,34,35]. Natriuretic peptides (NPs), like that described in Table 2, have profound effects on the cardiovascular system [36]. Their structures are promising for the design of novel medicines with vasodilator, natriuretic and diuretic properties [37]. The physiological role of the Kunitz/BPTIs (bovine pancreatic trypsin inhibitors) in snake venom is not completely understood. It was proposed that they participate in the processes of coagulation, fibrinolysis and inflammation through protease inactivation [38]. The sequence analysis of the two Kunitz inhibitors, isolated from the venom of*N. scutatus*, showed that the reactive site residues are different in the two peptides. The P1 residue is considered to be the most critical site defining the specificity and inhibitory interaction with serine proteases [39,40]. The amino acid sequence of the Kunitz/BPTI inhibitors, isolated from *N. scutatus* venom, was found also by analyzing the transcriptome of the *Notechis scutatus scutatus* venom gland [10]. The sequences are compared in Figure 4B. The amino acid residues at position P1 of the two inhibitors are different: a positively charged Arg in the first peptide and a neutral Asn in the second (Figure 4B). Other amino acids at the primary binding loop (P3P2P1P1′P2′P3′P4′) can also interact with proteolytic enzymes [41]. The sequences of this loop in the two inhibitors are quite different, PCRGILH and TGPCNRNTQ, respectively. Previous studies have shown that at least one of the residues at P2′ or P4′ should have a basic histidine residue to form a complex with kallikrein ([42] and the references therein). The first polypeptide has a histidine at the P4′ position (Figure 4B), and this explains its inhibitory activity towards kallikrein, as we have observed (Table 2). The second has a glutamine at the same position (Figure 4B), and we did not observe the inhibition of kallikrein by this peptide. It can be concluded that the different amino acid residues at the primary binding loop of both Kunitz/BPTI inhibitors are responsible for their inhibitory specificity towards serine proteases. A search in the UniProt database indicated that the sequence motif, NRN, at P1P1′P2′ position was found only in the *Notechis scutatus scutatus* venom Kunitz inhibitor, a species related to*N.scutatus*. The observed high affinity of the isolated polypeptide inhibitor towards kallikrein could be used for the design of specific inhibitors of plasma kallikrein to treat diseases, such as hereditary angioedema and septicemia, related to the increased blood levels of this enzyme [43]. 

## 4. Experimental Section

### 4.1. Material

Crude venoms from *N. m. mossambica* and *N.scutatus* from Kangaroo Island were a kind gift from Venom Supplies, Australia. The venoms were filtered to remove potential mucosal contaminants, lyophilized and stored at −20 °C until required.

### 4.2. Fractionation of the Crude Venoms and Purification of Peptides

The crude venoms were fractionated by size-exclusion chromatography (SEC). Twenty five milligrams of *N. m. mossambica* and 40 mg of the *N.scutatus* venom were dissolved in 0.1 M ammonium acetate, pH 5.0, and applied on a Superdex G-75 column previously equilibrated with the same buffer. The chromatography was performed using the same mobile phase at a flow rate of 1 mL/min. The UV absorbance of the eluate was monitored at 220 and 280 nm. Fractions were collected and analyzed by SDS-PAGE in 15% glycine gel or 18% Tris/Tricine gel. The gels were stained with Coomassie Blue. The collected fractions were further separated and peptides purified by liquid chromatography as follows: The peptides of Peak 5 (Figure 1A) were separated on a Resource S-1 mL cation-exchange column using NaCl gradient, 0 to 50% B, at a flow rate of 1 mL/min. Buffer A was 0.05 M sodium acetate, pH 5.5, and Buffer B was 0.05 M sodium acetate containing 1.0 M NaCl, pH 5.5. The peptides of Peaks 8 and 9 (Figure 1A) were separated by RPC (Reverse Phase Chromatography) using a C18 column (150/4.6). A linear gradient of 0%–75% B was used for elution at a flow rate of 0.8 mL/min. Zero-point-zero-five percent formic acid was used as Solvent A and acetonitrile as Solvent B. Peak 4 (Figure 1B) was fractionated on a SOURCE™ 5RPC column (150/4.6) by a linear gradient of 5%–75% at a flow rate of 1 mL/min for 55 min. Solvent A was 0.1% formic acid, and Solvent B was acetonitrile. 

Peptides inhibiting ACE were isolated by the filtration of the fractions of Peak 4 (Figure 1B) through a 3-kDa Amicon membrane. Peptides of the filtrate were further fractionated on a Chromolith C18 column (100/4.6) with a linear gradient of 0.3% B to 60% B, at a flow rate of 1 mL/min for 40 min. Zero-point-two percent formic acid was used as Solvent A and acetonitrile as Solvent B.

### 4.3. Enzyme Inhibition Assays

The inhibitory activity of snake venom peptides was tested towards trypsin, thrombin, recombinant subtilisin (Stmpr1), chymotrypsin, factor Xa, plasmin, plasma kallikrein and the angiotensin I converting enzyme. All enzymes were purchased from Sigma, except Stmpr1 (which was supplied in terms of collaboration). All substrates were purchased from Bachem. The measurements were performed at room temperature applying a micro-plate reader (infinite 200 PRO^®^, TECAN). Twenty microliters of each fraction were incubated with the respective protease for 15 min, and the residual protease activity was determined using the corresponding substrate. Bz-Phe-Val-Arg-pNA-HCl was the substrate for trypsin and thrombin, Suc-Ala-Ala-Pro-Phe-pNA for subtilisin and chymotrypsin, Z-D-Arg-Gly-Arg-pNA-HCl for factor Xa, Bz-Arg-pNA for plasmin and Bz-Pro-Phe-Arg-pNA for kallikrein. The release of 4-nitroaniline (pNA) was monitored at 405 nm. The inhibition of the 20S proteasome was measured with Z-Gly-Gly-Leu-7-amido-4-methyl-coumarin (AMC) as a substrate. The release of the 7-amido-4-methyl-coumarin (AMC) group was followed spectrofluorometrically at λ_ex_ = 360 nm and λ_em_ = 480 nm. The angiotensin I-converting enzyme activity in the presence of venom peptides was determined by a fluorescence resonance energy transfer assay using Abz-Phe-Arg-Lys(Dnp)-Pro-OH as a substrate [44]. The hydrolysis of the peptide bond between the fluorescent group (*o*-aminobenzoic acid, Abz) and the quencher (2,4 dinitrophenyl group, Dnp) generates a fluorescence emission, which was followed at λ_ex_ = 320 nm and λ_em_ = 420 nm. 

### 4.4. Mass Spectrometric Analyses

Snake venom peptides with molecular masses in the range 3.5–7 kDa were subjected to tryptic digestion in the presence of 6 M urea. One-point-three microliters of 100 mM dithiothreitol were added to 50 µL of the urea solution containing the respective peptide, and the mixture was incubated at 60 °C for 10 min. Then, 1.3 μL of 300 mM iodoacetamide were added, and the solution was incubated for 30 min in the dark. Four hundred twenty five microliters, 100 mM NaHCO_3_, pH 8.3, and 5 µL (0.25 µg/µL) of trypsin solution (sequencing grade modified trypsin; Promega, Madison, WI, USA) were added to the peptide solution, and the mixture was incubated at 37 °C for 16 h. The reaction was quenched by the addition of formic acid to a final pH of 3.0. Peptide identification was performed on a ESI-ion trap mass spectrometer (LC/MSD trap XCT Ultra, Agilent Technologies, Palo Alto, CA, USA), equipped with HPLC chip cube technology (Agilent Technologies). The HPLC-chip (large capacity chip, Agilent Technologies) integrating two on-chip columns, an enrichment column (internal volume: 160 nL) and a separation column (150 mm, both 5 µm Zorbax 300 SB-C18 material), as well as a nanospray emitter. A capillary pump attached to a microwell plate autosampler was used for the sample injection. Gradient elution was performed with a Nanoflow LC pump (1100 series Nanoflow LC System for MS, Agilent Technologies). Agilent ChemStation and MSD Trap Control software were used for system control and data acquisition. Mobile phase gradients consisted of 0.2% formic acid (Solvent A) and acetonitrile (Solvent B). For the analysis of tryptic bovine serum albumin (BSA) peptides, 1 µL of a 100 fmol/µL sample in Solvent A was injected. Samples were loaded from the autosampler onto the enrichment column of the HPLC-chips with a mobile phase of 2% Solvent B at a flow rate of 3 µL/min. The separation was performed with a gradient of 2%–40% Solvent B in 40 min at a flow rate of 400 nL/min. Data were acquired in the positive ion mode, applying a voltage of −1.8 kV at the electrospray inlet capillary, a nitrogen drying gas flow of 4 L/min and a temperature of 325 °C at the transfer capillary for desolvation. The mass spectrometer was operated in a data-dependent mode in which the three most intense ions in the precursor ion scan were subjected to subsequent automated MS/MS. Doubly charged ions were preferably selected for fragmentation. The isolation width was set to 4 m/z and the MS/MS fragmentation amplitude to 1.25 V. Active exclusion was enabled after three cycles of MS/MS; the precursor ion was released from the exclusion after 1 min. The generic ﬁles for database searching were generated by Data Analysis software for 6300 Series Ion Trap LC/MS version 3.4; for precursor ion selection, a threshold of 100,000 and a retention time window of 0.5 min were applied, and the absolute number of compounds was restricted to 1000 per MS/MS experiment. Protein identification was performed with a Mascot online search [45] MS/MS datasets were used to search the spectra against the subset “other lobe-finned fish and tetrapod clade” of the Swiss-Prot database [46]. Carbamidomethyl (C) and oxidation (M) were fixed as variable modifications, and the MS/MS tolerance was set to ± 0.6 Da.

MALDI-TOF and MALDI-TOF-TOF analyses were performed on an ultrafleXtreme instrument (BrukerDaltonics, Bremen, Germany) equipped with a smartbeam-II laser with a repetition rate of 1 kHz. The spots were measured automatically by an autoXecute method within the FlexControl software (version 3.3). In MS mode, spectra were acquired in a mass range of m/z 800–3500 by summing up 1500 laser shots. Mass spectra were externally calibrated using a peptide calibration mixture II (BrukerDaltonics) within the m/z range between m/z 1046 and m/z 3147. For acquiring MS/MS spectra, up to 20 precursor masses were selected meeting the following criteria: peak intensity above 500 and a signal-to-noise ratio greater than 10. The primary mass choice range was set to m/z 1000–2500, while the secondary mass choice range was m/z 800–3500. MS/MS spectra were obtained with laser-induced dissociation (LID) summing up 2000 laser shots. The spectra were processed using FlexAnalysis software (version 3.3). Samples were spotted on a MALDI target plate (MTP Anchor Chip 384, BrukerDaltonics) using cyano-4-hydroxycinnamic acid matrix. Samples mixed with 2,5-dihydroxybenzoic acid matrix were spotted on ground steel target (BrukerDaltonics). FlexAnalysis (version 3.3, BrukerDaltonics) was used to process the MS spectra. Further data analysis was performed using BioTools (Version3.2, BrukerDaltonics) and Mascot Inhouse Search. Mascot [45] version 2.1.03 was used to analyze the spectra against the subset “other lobe-finned fish and tetrapod clade” of the Swiss-Prot database.

ESI-TOF-MS analysis was performed on an LC/ESI-TOFMS instrument (Agilent 6224 ESI-TOF, Agilent Technologies). The injection volume was 5 µL for each sample. Data acquisition was carried out using the Agilent MassHunter software (version B.03.01) in positive ESI mode. The parameters were: gas temperature, 325 °C; drying gas flow, 10 L/min; nebulizer gas, 15 psig; VCap, 4000 V; fragmentor voltage, 200 V; Skimmer, 65 V; Oct 1 RF Vpp 750 V; mass range, 110–3200 m/z; permanent lock mass calibration (m/z 121.0509 and m/z 922.009); acquisition rate 1, 03 spectra/s.

ESI-QTOF analysis was performed on a Q-TOF-2 electrospray mass spectrometer (Waters, Eschborn, Germany). The experiments were carried out in the positive ion mode (ES (+)). Nanoflow capillaries were drawn and coated with gold (done in-house). The capillaries were loaded with a 2-μL sample, and low pressure nitrogen gas was used to initiate the flow through the capillary. The capillary tip was set to a potential of 0.66 kV, and the cone voltage was set to 34 V. The source temperature was 20 °C. For MS/MS experiments using collision-induced dissociation (CID) experiments, ions were selected within a precursor mass window of ± 1 Da in the quadrupole analyzer and fragmented in the collision cell using a collision gas (Ar) and collision energies of 27 to 30 eV. The cycle time was about 1.1 s with a scan duration of 1 s. Raw data were acquired and analyzed using the software, MassLynx 3.5 (Micromass, Manchester, UK). MS/MS spectra were automatically processed with the micromass charge state de-encryption algorithm (MaxEnt-3). Glycan analysis was performed manually.

The MALDI-TOF, MALDI-TOF-TOF MS and ESI-TOF experiments were performed under the supervision of Dr. M. Trusch at the Department of Organic Chemistry, University of Hamburg. ESI-QTOF analysis and analysis of the tryptic digest were performed under the supervision of Prof. Dr. H. Schlüter at University Medical Centre Hamburg-Eppendorf, Hamburg.

## 5. Conclusions

The comparative analyses of the peptide composition of two elapid venoms, one from Elapinae, the other from Acanthophiinae, indicates that both contain pharmacologically interesting peptides. Our data also indicate that there are sub-family specificities in the peptide composition of these venoms. The *N.scutatus* venom contains Kunitz-type inhibitors, which were not detected in the *Naja mossambica mossambica* sample. Three-finger toxins were found in both venoms. However, the *N. scutatus* venom contains only neurotoxin-type three-finger peptides and lacks cytotoxin-type three-finger peptides, which constitute the main peptide composition of *N. m. mossambica* venom. Interestingly, hypotensive peptides are present in both venoms, indicating that during evolution, these peptides were conserved, due to their essential functions in snake venoms**. **Although the two Kunitz/BPTI inhibitors isolated from *N. scutatus*venom are homologues to each other, they display differences in the reactive bond loop residues, suggesting that nature has engineered these peptides to perform a variety of functions by incorporating subtle mutations at exposed binding loops. The data obtained indicate that peptides are important constituents of snake venoms and functions, by impairing or affecting vital components of the prey’s homeostasis. Among the identified molecules, several can, in principle, possess potential pharmacological applications and can serve as tools or as prototypes for drug design studies, with the advantage that unlike other active components of the venom, peptides are much easier to synthesize and less prone to inducing an immune response.

## Abbreviations

ACE: angiotensin-converting enzyme;
CTX: cardiotoxin;
BPP: bradykinin-potentiating peptide;
NP: natriuretic peptide;
3-F: three-finger peptide
